# The relationship between seasonality, latitude and tuberculosis notifications in Pakistan

**DOI:** 10.1186/s12879-021-05899-x

**Published:** 2021-02-25

**Authors:** Mohsin F. Butt, Sidra Younis, Zhenqiang Wu, Syed H. Hadi, Abdullah Latif, Adrian R. Martineau

**Affiliations:** 1 The Wingate Institute of Neurogastroenterology, Centre for Neuroscience, Trauma and Surgery, The Blizard Institute, Barts and The London School of Medicine and Dentistry, Queen Mary University of London, 26 Ashfield Street, Whitechapel, London, E1 2AJ UK; 2 Department of Respiratory Medicine, Royal Free Hospital, Royal Free NHS Foundation Trust, Pond Street, Hampstead, London, NW3 2QG UK; 3 Department of Biological Sciences, National University of Medical Sciences (NUMS), Abid Majeed Road, Rawalpindi, Pakistan; 4 Institute of Population Health Sciences, Yvonne Carter Building, Barts and The London School of Medicine and Dentistry, Queen Mary University of London, 58 Turner Street, Whitechapel, London, E1 2AB UK; 5 Department of Geriatric Medicine, The University of Auckland, Auckland, New Zealand; 6 National Tuberculosis Control Programme, Islamabad, Pakistan

**Keywords:** Tuberculosis, Pulmonary, Respiration disorders, Public health

## Abstract

**Background:**

Pakistan ranks amongst the top 20 highest burden tuberculosis (TB) countries in the world. Approximately 369,548 cases of TB (all forms) were notified in 2018, with an estimated incidence of 265 per 100,000 people per year. In other settings, TB has been shown to demonstrate seasonal variation, with higher incidence in the spring/summer months and lower incidence in the autumn/winter; the amplitude of seasonal variation has also been reported to be higher with increasing distance from the equator.

**Methods:**

Notifications of newly-diagnosed pulmonary and extrapulmonary TB cases were obtained for 139 districts in Pakistan from 2011 to 2017. Data were provided by the Pakistan National TB Control Programme, Islamabad, Pakistan. Statistical analyses were performed to determine whether there was seasonal variation in TB notifications in Pakistan; whether the amplitude of seasonal variation in TB notifications varied according to latitude; whether the amplitude of seasonal variation of TB in Pakistan differed between extrapulmonary TB vs. pulmonary TB. To assess the quarterly seasonality of TB, we used the X-13-ARIMA-SEATS seasonal adjustment programme from the United States Census Bureau. The mean difference and corresponding 95% confidence intervals of seasonal amplitudes between different latitudes and clinical phenotype of TB were estimated using linear regression.

**Results:**

TB notifications were highest in quarter 2, and lowest in quarter 4. The mean amplitude of seasonal variation was 25.5% (95% CI 25.0 to 25.9%). The mean seasonal amplitude of TB notifications from latitude 24.5°N- < 26.5°N was 29.5% (95% CI 29.3 to 29.7%) whilst the mean seasonal amplitude of TB notifications from latitude 34.5°N - < 36.5°N was 21.7% (95% CI 19.6 to 23.9%). The mean seasonal amplitude of TB notifications across Pakistan between latitudes 24.5°N to 36.5°N reached statistically significant difference (*p* < 0.001). The amplitude of seasonal variation was greater for extrapulmonary TB (mean seasonal amplitude: 32.6, 95% CI 21.4 to 21.8%) vs. smear positive pulmonary TB mean seasonal amplitude: 21.6, 95% CI 32.1 to 33.1%), *p* < 0.001.

**Conclusion:**

TB notifications in Pakistan exhibit seasonal variation with a peak in quarter 2 (April–June) and trough in quarter 4 (October–December). The amplitude of seasonality decreases with increasing latitude, and is more pronounced for extrapulmonary than for pulmonary TB.

**Supplementary Information:**

The online version contains supplementary material available at 10.1186/s12879-021-05899-x.

## Background

Tuberculosis (TB) is an infectious disease caused by bacteria of the *Mycobacterium tuberculosis* complex*,* which can spread by small airborne droplets [1]. TB was the leading cause of death from a single infectious pathogen in 2016 [2] and is highly endemic in Pakistan. According to 2018 figures from the World Health Organisation, Pakistan ranks  amongst the top 20 highest burden TB countries in the world, with an estimated incidence of 265 per 100,000 people per year [3].

In numerous setting worldwide, the incidence of TB has been shown to have seasonal variation, with peak levels during the summer months [4–7] and trough notification rates in the winter season [8–11]. To our knowledge, Khaliq et al. [12] have been the only group to explore the seasonality of TB in Pakistan. The group studied the seasonal variation in newly diagnosed pulmonary TB cases notified to the directly observed therapy short course of the national TB programme in a single district (Lahore) from 2006 to 2013. In this study, the seasonal adjusted factor showed peak TB notifications in the second quarter of the year (April to June) in Lahore, Pakistan [12].

The pattern of TB seasonality is the reverse other respiratory diseases [5], and the cause of this paradoxical trend is currently unelucidated. One dominant hypothesis implicates the seasonal variation in serum vitamin D levels [13]. The cutaneous synthesis of vitamin D, the “sunshine vitamin” [14], occurs in the presence of ultraviolet B-radiation (UVB). Vitamin D has been shown to modulate immune function [15], particularly macrophage activity. As such, it is hypothesised that the low serum vitamin D levels during winter months may compromise immune function and, following a temporal lag, result in latent TB reactivation [5, 16]. The vitamin D hypothesis of TB seasonality is supported by our cross-sectional study conducted in South Africa, in which we reported a reciprocal seasonal relationship between reduced serum vitamin D concentration and increased TB notifications [17]. Moreover, we have previously shown that in vivo vitamin D supplementation enhances immunity to mycobacteria both in healthy people [18] and in a genetically defined subgroup of patients with active TB [15].

The earth’s surface receives more solar radiation at low latitudes i.e. regions closer to the equator [19], and this has been shown to be related to the likelihood of vitamin D deficiency at a population level [20]. As such, it would be reasonable to hypothesise that the seasonal amplitude of TB notifications in Pakistan may differ depending on distance from the equator, with more pronounced amplitude of seasonal variability in regions with less sunlight [11, 21].

The seasonality of TB may vary depending on disease phenotype i.e., pulmonary or extrapulmonary TB. For instance, in India, extrapulmonary TB has been shown to have greater seasonal variation compared to pulmonary TB [11]. Moreover, one study [22] investigating the relationship between vitamin D and disease phenotype reported a specific association between vitamin D deficiency and the development of extrapulmonary tuberculosis, raising the possibility that the amplitude of seasonal variation might be greater for extrapulmonary TB than for pulmonary TB. As such, it would be reasonable to hypothesise that the seasonal amplitude of TB notifications in Pakistan would be greater for  extrapulmonary TB compared to pulmonary TB.

Hitherto, no study has investigated the relationship between seasonality, latitude and TB notifications in Pakistan, which could guide public health interventions and policy decision making. We therefore conducted the current study, with three aims: to determine whether there is seasonal variation in the notifications of active TB in Pakistan; to determine whether the amplitude of seasonal variation of active TB varies according to latitude; and to determine whether the amplitude of seasonal variation of TB in Pakistan differs between extrapulmonary vs. pulmonary TB.

## Methods

Data for newly diagnosed smear positive pulmonary and extrapulmonary TB cases were obtained for 139 districts (see supplementary Table 1) in Pakistan from 2011 to 2017. Data were provided by the Pakistan National TB Control Programme, Islamabad, Pakistan. Data were provided quarterly: quarter 1 (January–March), quarter 2 (April–June), quarter 3 (July–September), quarter 4 (October–December). In the Pakistan National TB Control Programme, smear positive pulmonary TB is diagnosed using sputum smear microscopy, whereby two sputum samples are collected under a microscope for the presence of acid fast bacilli, whilst extrapulmonary TB is diagnosed using clinical methods or histopathology [23]. The latitudes of each of the 139 districts were obtained from the following website: https://latitude.to/.

The quarterly seasonality of TB was assessed using the X-13-ARIMA-SEATS seasonal adjustment software from the United States Census Bureau [16]. The quarterly time series was decomposed into the trend component, the seasonal component and the irregular component (R package: ‘seas’). A decomposition of monthly notification was conducted for extrapulmonary and pulmonary TB. The mean peak month, trough month and annual seasonal amplitude with 95% confidence intervals (CIs) were calculated if identifiable seasonality was assessed by WO-test [24]. The annual seasonal amplitude was calculated from isolated seasonal factor and defined as the fraction with the numerator representing the peak-to-trough different between the months with the highest and the lowest case counts and the denominator as the mean case counts for that year [16]. The mean difference and corresponding 95% confidence intervals of seasonal amplitudes between different latitudes and type of TB were estimated using linear regression. Statistical analyses were performed using R version 3.6.3. A *p* value< 0.05 was considered statistically significant.

Data from the Pakistan National TB Control Programme were aggregated without any personal information, hence informed consent was not required. The study was approved by the Institutional Review Board of the National University of Medical Sciences, Rawalpindi, Pakistan (reference number: NUMS/PVC-19/R&D/ORIC/IRB&EC).

## Results

**Fig. 1:**
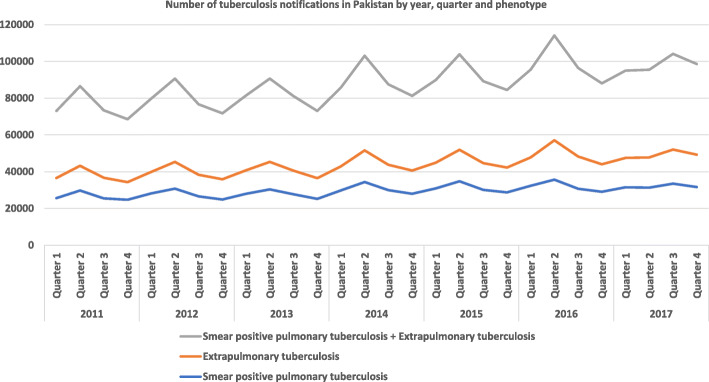
Number of tuberculosis notifications in Pakistan by quarter, year and phenotype. Original figure. No permissions required

**Fig. 2:**
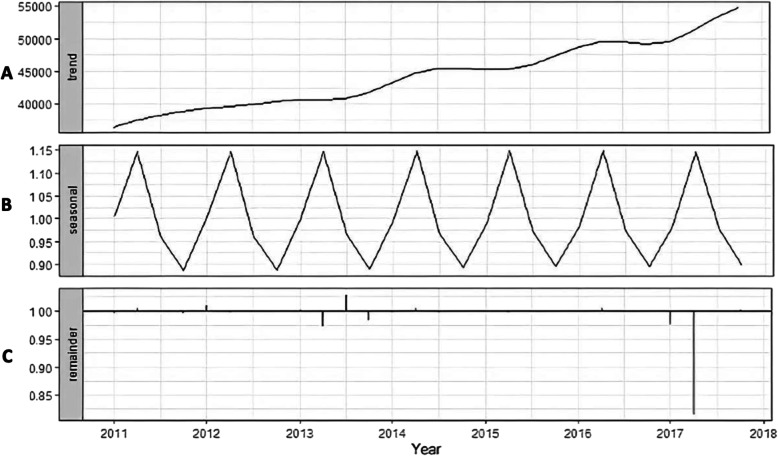
X-13-ARIMA seasonal decomposition of monthly notifications of active tuberculosis (extrapulmonary and smear positive pulmonary TB, see Fig. 1) in Pakistan from 2011 to 2017: trend cycle (**a**), seasonal component (**b**) and remainder component (**c**). Definitions: *trend cycle* - the component that represents variations of low frequency in a time series, the high frequency fluctuations having been filtered out; *seasonal component* - that part of the variations in a time series representing intra-year fluctuations that are more or less stable year after year with respect to timing, direction and magnitude; *remainder component* - the residual time series after the trend-cycle and the seasonal components (including calendar effects) have been removed. It corresponds to the high frequency fluctuations of the series. Original figure. No permissions required

A total of 12,295,88 newly diagnosed pulmonary and extrapulmonary TB cases were notified to the Pakistan National TB Programme for 139 districts from 2011 to 2017. The notification of newly diagnosed pulmonary and extrapulmonary TB cases are presented in Fig. 1**.** The original time series of monthly notifications of active TB cases displays seasonal fluctuation. The X-13-ARIMA-SEATS seasonal adjustment method was then used to decompose the original time series into three components: trend-cycle (Fig. 2a), seasonal component (Fig. 2b), and remainder component (Fig. 2c). The trend-cycle reflects the long-term progression of the time series where the high frequency fluctuations have been filtered out [25]. Our trend-cycle shows a general upward trend of TB notifications from 2011 to 2017. The seasonal component (referred to as the seasonality of the time series) is that part of the variations in a time series representing intra-year fluctuations that are more or less stable year after year with respect to timing, direction and magnitude [26]. Our seasonal component did not change over time. The remainder (irregular) component includes random fluctuations, abnormal values, and other irregular factors [27]. Of note in our remainder component is the marked reduction in the notifications of TB in quarter 2 of 2018, but the reasons for this are unclear. Analysis of the isolated seasonal component revealed that the annual seasonal amplitude for TB notifications was 25.5% (95% CI 25 to 25.9%), suggesting an annual mean of 25.5% additional cases of TB diagnosed in the quarter two (April – June) compared to quarter four (October – December).

**Fig. 3:**
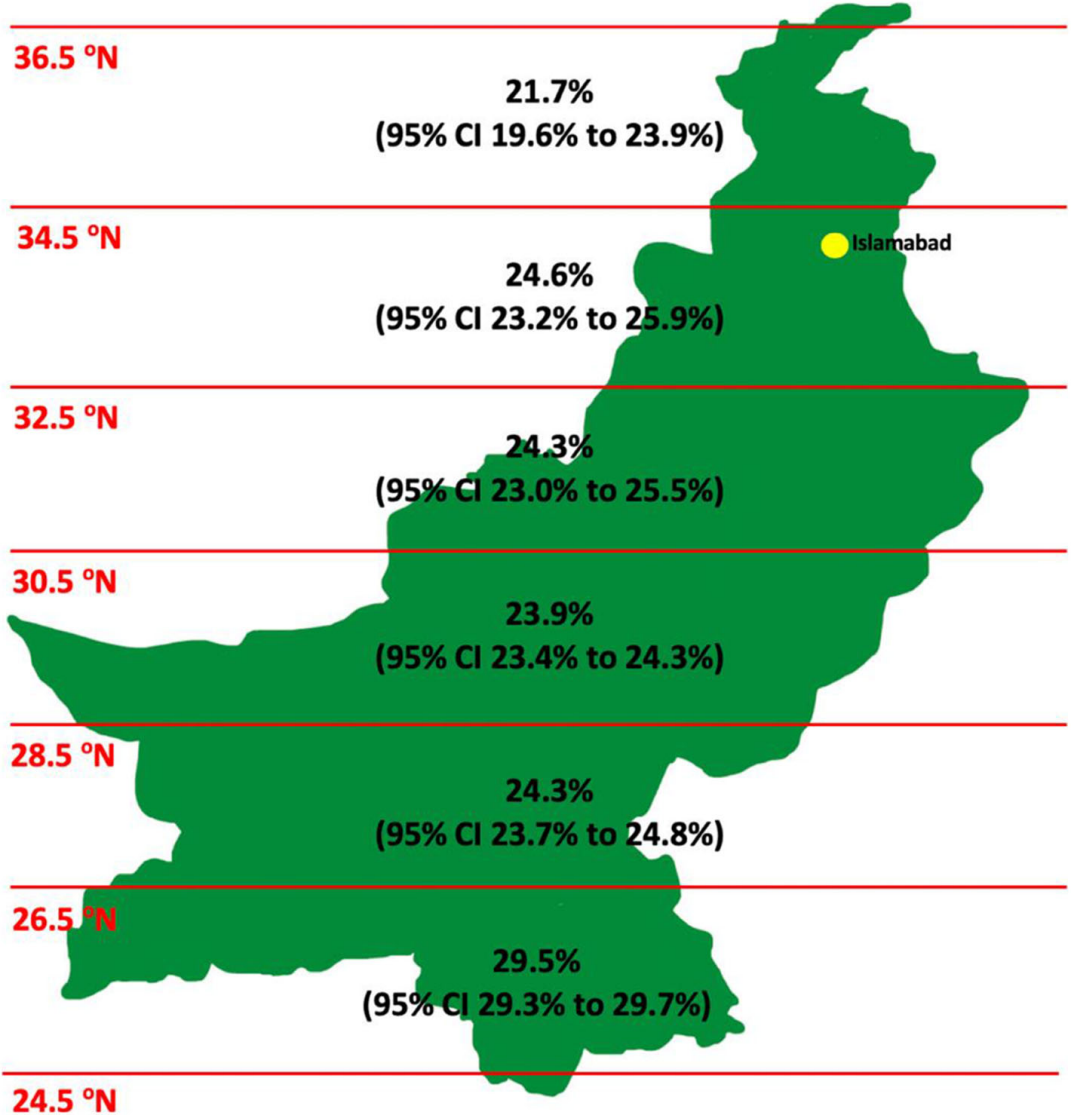
The mean seasonal amplitude of TB notifications from 24.5°N to 36.5°N across Pakistan. The 95% confidence interval (CI) is described. The location of Islamabad – the capital city of Pakistan – is highlighted in yellow. Original figure. No permissions required

Table 1 shows the effect of latitude on the seasonal variation of tuberculosis notifications and the difference in the mean seasonal amplitude of extrapulmonary and pulmonary TB. Increasing distance from the equator was associated with a reduction in mean seasonal amplitude of TB notifications (*p* < 0.001). At districts closer to the equator (latitude range 24.5°N - < 26.5°N), the mean seasonal variation of TB was 25.5% (95% CI 25.0 to 25.9%) whilst districts further away from the equator (latitude range 34.5°N - < 36.5°N) had a mean seasonal variation of TB of 21.7% (95% CI 19.6 to 23.9%). The mean seasonal amplitude for extrapulmonary TB notifications was 11% (95% CI 10.6 to 11.5%) higher than for pulmonary TB, *p* < 0.001. The mean seasonal amplitude of TB notifications across Pakistan (latitude ranges 24.5°N - 36.5°N) is graphically illustrated in Fig. 3.

**Table 1:** The relationship between seasonality, latitude and tuberculosis phenotype in Pakistan. Original table. No permissions required

	Peak /Trough quarter	Mean seasonal amplitude (%), 95% CI	Mean difference (%), 95% CI, p	Type III test for group difference
**Overall**	Q2/Q4	25.5 (25.0–25.9)	NA	
**Latitude**				**< 0.001**
24.5- < 26.5	Q2/Q4	29.5 (29.3–29.7)	7.7 (6.3, 9.2), < 0.001	
26.5- < 28.5	Q2/Q4	24.3 (23.7–24.8)	2.5 (1.1, 4.0), < 0.001	
28.5- < 30.5	Q2/Q4	23.9 (23.4–24.3)	2.1 (0.7, 3.6), 0.004	
30.5- < 32.5	Q2/Q4	24.3 (23.0–25.5)	2.5 (1.1, 4.0), < 0.001	
32.5- < 34.5	Q2/Q4	24.6 (23.2–25.9)	2.8 (1.4, 4.3), < 0.001	
34.5- < 36.5	Q2/Q4	21.7 (19.6–23.9)	0.0 (reference)	
**Type of TB**				**< 0.001**
Pulmonary TB	Q2/Q4	21.6 (21.4–21.8)	0.0 (reference)	
Extrapulmonary TB	Q2/Q4	32.6 (32.1–33.1)	11.0 (10.6, 11.5), < 0.001	

Notes: the mean annual seasonal amplitude was calculated from the seasonal component as the annual difference between the peak and trough as a proportion of the annual mean case count

## Discussion

To our knowledge, we are the first group to investigate the seasonality of tuberculosis across Pakistan and the effect of latitude on this relationship. We report seasonal variation of TB notification across 139 districts in Pakistan, with a peak in TB notifications in quarter two (April–June) and trough in quarter four (October–December). The amplitude of seasonal variation in TB notifications was greater in regions closer to the equator. Seasonal variation in notifications was also more pronounced for extrapulmonary than for pulmonary TB.

The results of our study are consistent with those of a smaller study looking at the seasonality of TB in a single district, Lahore, Pakistan, in which investigators showed a peak in pulmonary TB cases in the second quarter of the year [12]. Several studies [9, 11, 16, 21] have explored the relationship between distance from the equator and seasonal variation of TB, and it is plausible that the seasonality of TB is more pronounced in areas where UV exposure, and therefore cutaneous vitamin D synthesis, is low [11, 21]. Contrary to a study published in India [11], which showed that northern areas have greater seasonal variation than those in central southern regions, our study shows reduced seasonal variation in regions further from the equator. Our results are consistent with a study investigating TB seasonality in Xinjiang Province, northwest China, in which the amplitude of TB seasonality was greater in southern areas (34°N-42°N) than eastern and northern regions (43°N-48°N); although, the difference was not statistically significant [16]. In the USA, the amplitude of TB seasonality has not been shown to vary by latitude [9].

Several hypotheses have put forward to explain the relationship between seasonality and tuberculosis, including winter indoor crowding [28–30], seasonal variation of other respiratory infectious diseases [31], seasonal variation in dietary nutrient intake [28] and fluctuations in cutaneous synthesis of vitamin D [16, 28]. The degree to which each of these variables influence seasonal variation of TB is hitherto unclear. In India, TB has been shown to have a greater amplitude of seasonal variation than pulmonary TB [11]. In a UK setting [22], vitamin D deficiency has been shown to be strongly and specifically associated with extrapulmonary TB at the time of diagnosis, and doubling of serum vitamin D concentration may confer a significantly reduced risk of extrapulmonary disease. Overall, this suggests that extrapulmonary TB is more sensitive to changes in vitamin D concentration, which may explain the greater seasonal variation in extrapulmonary than pulmonary TB in our study. 

Key strengths of this study are that we have pooled outcomes of pulmonary and extrapulmonary TB to maximise power. Moreover, we studied the seasonality of pulmonary and extrapulmonary TB separately and, consistent with our hypothesis, demonstrated that seasonal variation of extrapulmonary TB was more marked than for pulmonary TB. Data were analysed on a district level, which maximized their granularity. However, our analysis has limitations. Data were not collected on a monthly basis; hence it was not possible for us to identify the specific month/s of the year in which TB peaked or nadired. Data on sex and age were not available for each year, hence we were not able to study whether these variables affected the amplitude of seasonal variation [9] nor if different age groups or sexes had a different peak/trough of TB notifications [32]. Other important confounding variables that have been shown to affect the seasonality of TB in other settings include air pollution [33], HIV status [34] and climate parameters, such as rainfall and temperature [28]. We were also unable to collect data on the rural or urban nature of the different district. This would be important given a significant body of evidence demonstrates greater UV exposure in rural compared to urban areas [35–37], hence the seasonality of TB might be more pronounced in rural regions. Lastly, we only included smear-positive pulmonary TB patients in this study, hence the results are not applicable to all forms of pulmonary TB.

## Conclusion

TB notifications in Pakistan exhibit seasonal variation with a peak in quarter 2 (April–June) and trough in quarter 4 (October–December). The amplitude of seasonality decreases with increasing latitude, and is more pronounced for extrapulmonary than for pulmonary TB. The exact cause of the seasonal variation of TB notifications is unknown, but winter indoor crowding, poor UV exposure in winter, seasonal variation in immune function and coinfection with other seasonal pathogens may be responsible. This study may help to shape future public health responses, ensuring that governments can target public health interventions at specific times of the year to reduce TB transmission. Future analyses should ensure confounding variables, such as age, sex, air pollution, temperature and HIV status are controlled for.

## Supplementary Information

**Additional file 1: Table S1.** The districts in Pakistan analysed in this study. Original table. No permissions required.

## Data Availability

The data that support the findings of this study are available from the Pakistan National TB Programme, but restrictions apply to the availability of these data, which were used under license for the current study, and so are not publicly available. Data are however available from the authors upon reasonable request and with permission from the Pakistan National TB Programme.

## Abbreviations

TB: Tuberculosis
UVB: Ultraviolet B rays
