# Supplementary material for: The relationship between seasonality, latitude and tuberculosis notifications in Pakistan
Source: BMC Infect Dis. 2021 Feb 25;21:210. doi: 10.1186/s12879-021-05899-x (PMC7905850; doi:10.1186/s12879-021-05899-x)
Supplement: Supplementary file 1 — Additional file 1: Table S1. The districts in Pakistan analysed in this study. Original table. No permissions required. [file 12879_2021_5899_MOESM1_ESM.docx]

**Supplementary data**

**Supplementary table 1.** The districts in Pakistan analysed in this study. Original table. No permissions required.

| (TAY) Tando Allah Yar |
| --- |
| (TMK) Tando Muhmaad Khan |
| Abbottabad |
| Astore |
| Attock |
| Awaran |
| Badin |
| Bagh |
| Bahawal Nagar |
| Bahawalpur |
| Bajour |
| Bannu |
| Barkhan |
| Batagram |
| Bhakkar |
| Bhimber |
| Bolan |
| Buner |
| Chaghi |
| Chakwal |
| Charsada |
| Chiniot |
| Chitral |
| Dadu |
| Dera Ghazi Khan |
| Dera Ismail Khan |
| Diamer |
| Faisalabad |
| Fr Bannu/ Lakki |
| Fr Peshawar/ Kohat |
| Fr Tank/ Di Khan |
| Gawadar |
| Ghanche |
| Ghizer |
| Ghotki |
| Gilgit |
| Gujranwala |
| Gujrat |
| Hafizabad |
| Hangu |
| Haranai |
| Haripur |
| Havili |
| Hunza Nagar |
| Hyderabad |
| Jacobabad |
| Jaffarabad |
| Jamshoro |
| Jhal Magsi |
| Jhang |
| Jhelum |
| Jhelum Valley/Hattianbala |
| Kalat |
| Karachi Center (Karachi 18 Towns) |
| Karak |
| Kashmore |
| Kasur |
| Kech |
| Khanewal |
| Kharan |
| Kharipur |
| Khushab |
| Khuzdar |
| Khyber |
| Killa Abdullah |
| Killa Saifullah |
| Kohat |
| Kohistan |
| Kohlu |
| Kotli |
| Kurrum |
| Lahore |
| Lakki Marwat |
| Larkana |
| Lasbela |
| Layyah |
| Lodhran |
| Loralai |
| Lower Dir |
| Malakand |
| Mandi Baha Ud Din |
| Mansehra |
| Mardan |
| Mastung |
| Matiari |
| Mianwali |
| Mirpur |
| Mirpur Khas |
| Mohmand Agency |
| Multan |
| Musa Khail |
| Muzaffar Garh |
| Muzaffarabad |
| Nankana Sahib |
| Narowal |
| Naseerabad |
| Naushahro Feroze |
| Nawabshah |
| Neelum |
| North Waziristan |
| Noshiki (Nushki) |
| Nowshera |
| Okara |
| Orakzai |
| Pak Pattan |
| Panjgur |
| Peshawar |
| Pishin |
| Poonch/Rawalakot |
| Qamber Shahdad Kot |
| Quetta |
| Rahim Yar Khan |
| Rajan Pur |
| Rawalpindi |
| Sahiwal |
| Sanghar |
| Sargodha |
| Shangla |
| Sheikhupura |
| Shikarpur |
| Shirani |
| Sialkot |
| Sibi |
| Skardu |
| South Waziristan |
| Sudhnoti/Pallundry |
| Sukkar |
| Swabi |
| Swat |
| Tank |
| Tharparkar |
| Thatta |
| Toba Tek Singh |
| Umerkot |
| Upper Dir |
| Vehari |
| Washuk |
| Zhob |
| Ziarat |
